# Iron deficiency in sepsis patients managed with divided doses of iron dextran: a prospective cohort study

**DOI:** 10.1038/s41598-023-32002-y

**Published:** 2023-03-31

**Authors:** Piotr F. Czempik, Agnieszka Wiórek

**Affiliations:** 1 Department of Anaesthesiology and Intensive Care, Faculty of Medical Sciences in Katowice, Medical University of Silesia, Medyków 14, 40-752 Katowice, Poland; 2 Transfusion Committee, University Clinical Center of Medical University of Silesia in Katowice, Katowice, Poland

**Keywords:** Anaemia, Infection

## Abstract

Iron deficiency (ID) impairs hemoglobin (Hb) synthesis and immune function, both crucial for sepsis patients. We assessed the impact of iron dextran on reticulocyte (Ret) Hb equivalent (Ret-He) and Ret subpopulations in iron-deficient sepsis patients. In this prospective clinical study we enrolled patients with sepsis or septic shock with procalcitonin concentration > 0.5 ng/mL, diagnosed with ID based on Ret-He. Study subjects received divided doses of iron dextran until normalization of Ret-He. The study population included 35 subjects. The median Ret-He increase after 2 doses of iron dextran was 3.0 (IQR 1.9–6.1) pg (*p* < 0.01) with median time to normalization 4 (IQR 3–5) days. Although no change in Ret percentage [Me 1.5 (IQR 1.1–2.1) vs. Me 1.4 (IQR 1.1–2.4) %, *p* = 0.39] and number [Me 0.05 (IQR 0.04–0.07) vs. Me 0.05 (IQR 0.03–0.06) 10^6^/µL, *p* = 0.88] was noted, Ret subpopulations changed significantly (*p* for all < 0.01). Divided doses of iron dextran relatively quickly normalize Ret-He in iron-deficient sepsis patients. Changes in Ret subpopulations suggest increased erythropoietic activity. Further research is needed to explore the role of intravenous iron in this clinical setting.

## Introduction

Anemia is a common health problem, affecting up to 50% of population in certain geographical regions^1^. Iron deficiency is also the most common cause of anemia in general population^2^. Anemia may affect as many as 66% of patients at the time of admission to the intensive care unit (ICU), and almost all patients after 72 h of ICU hospitalization^3^. Anemia which develops during hospitalization is referred to as hospital-acquired anemia (HAA). The important cause of HAA in the ICU is withdrawal of blood for laboratory diagnostics^4,5^. Iatrogenic blood loss induced by laboratory diagnostics leads to iron deficiency (ID) if the amount of stored iron is inadequate.

Iron deficiency impairs both hemoglobin (Hb) synthesis and immune function, both particularly important in sepsis patients. Iron deficiency without anemia (ID) is a preclinical stadium for iron deficiency anemia (IDA), and already at this stage may have deleterious effects: deranged mitochondrial function; impaired synthesis of Hb, myoglobin, cytochromes, nitric oxide synthase; and impaired immune function^6^. Iron supplementation at the early stage may prevent the development of IDA. When anemia develops, it is associated with well documented complications: organ hypoxia, myocardial infarction, stroke, infection. Anemia in the critically ill patients is associated with prolonged weaning off the respirator, acute kidney injury, and increased mortality^7^. The causal treatment of IDA is iron replenishment. In IDA red blood cell (RBC) transfusion should be considered the treatment of last resort because RBC transfusion may lead to numerous complications, issue of particular importance in critically ill patients with sepsis or septic shock. Replenishment of iron at the early stage may prevent anemia and its consequences. Therefore sepsis patients with ID or IDA may benefit from intravenous supplementation of iron. Oral iron supplementation in septic patients is not effective as increased concentration of hepcidin in patients with systemic inflammatory response inhibits iron absorption from the gut though different molecular mechanisms^8,9^.

Due to their limitations tests other than iron studies and Hb concentration have to be used in order to diagnose ID and monitor the response to intravenous iron in sepsis patients. Both ferritin and transferrin are acute phase proteins and their concentrations will be deranged in sepsis, and they cannot be used for accurate ID or IDA diagnostics in this clinical scenario^10^. Disadvantage of Hb is a late response and the fact that its concentration may be affected by factors other than iron replenishment (e.g. continuous loss of blood for laboratory diagnostics). A new parameter that can be used for anemia classification in sepsis patients is reticulocyte Hb equivalent (Ret-He), which gives information on functional availability of iron for erythropoiesis in the last 3–4 days, what corresponds to a Ret lifespan^11^. Reticulocyte Hb equivalent also quickly normalizes with iron therapy and can be used for monitoring of therapy with intravenous iron^12^. Modern hematology analyzers, apart from Ret-He, give information on Ret subpopulations: immature Rets fraction (IRF), low fluorescence ratio Rets (LFR), medium fluorescence ratio Rets (MFR), high fluorescence ratio Rets (HFR). The degree of fluorescence emission is proportional to the amount of RNA in the Ret, therefore the higher fluorescence emission the more immature subpopulation of Rets. Determination of Ret subpopulations may provide information on bone marrow erythropoietic activity and be also of use in monitoring the response to intravenous iron in sepsis patients.

The aim of the study was to assess the impact of intravenous iron on Ret-He and Ret subpopulations in iron-deficient patients with sepsis or septic shock (the most recent definitions), with an elevated procalcitonin (PCT) concentration for improved sepsis diagnostic accuracy, hospitalized in the ICU.

## Methods

### Study subjects

**Figure 1:**
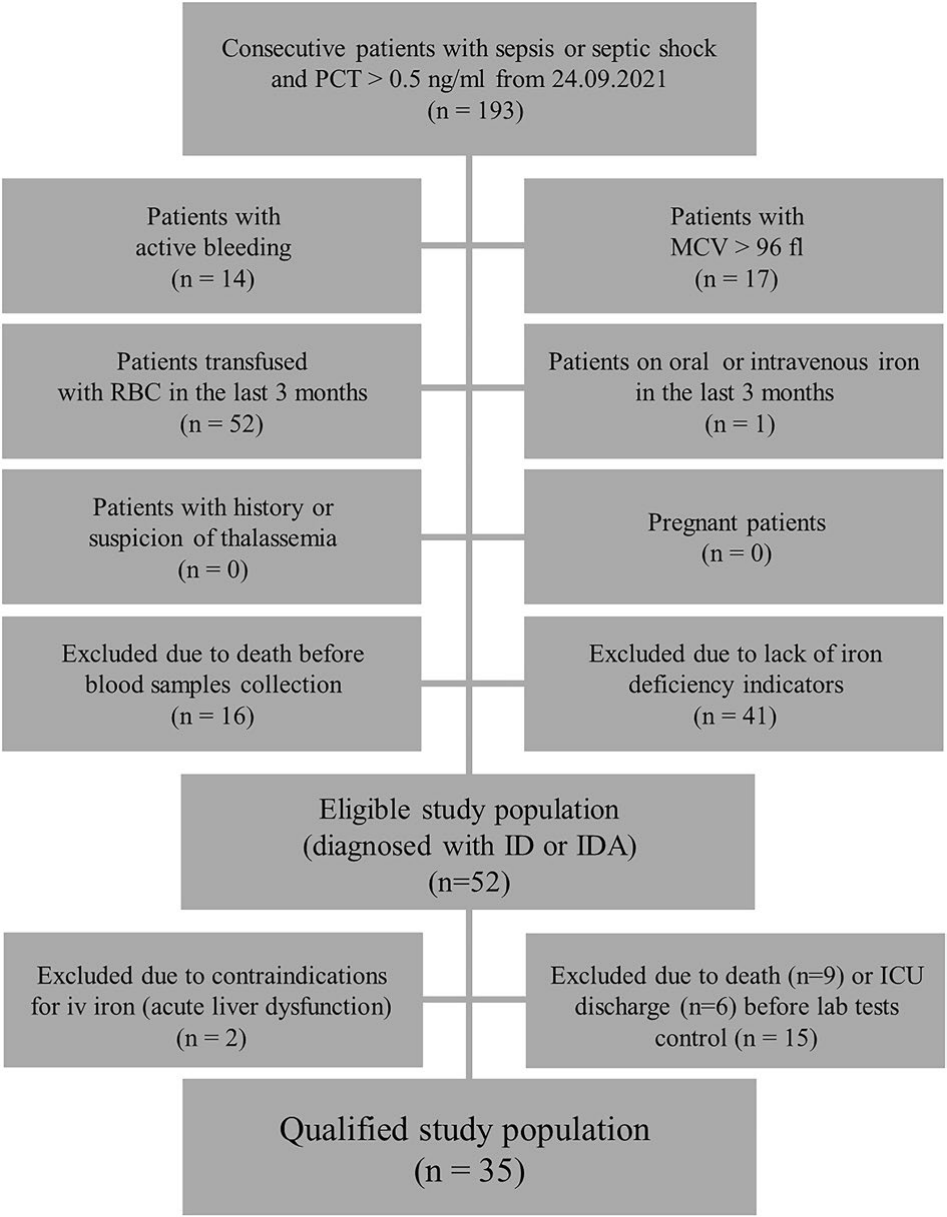
The study flow chart.

We conducted a prospective analysis of consecutive sepsis patients hospitalized in a 10-bed mixed medical-surgical ICU in a tertiary care teaching hospital. The enrollment period was from September 2021 to June 2022. The inclusion criteria were: sepsis or septic shock with PCT concentration > 0.5 ng/mL. Sepsis and septic shock were diagnosed using the third international definition^13^. Although PCT determination is not required to establish a diagnosis of sepsis or septic shock according to the most recent Surviving Sepsis Campaign guidelines^14^, it is sometimes difficult to distinguish organ dysfunction caused by sepsis from organ dysfunction due to other causes in the ICU. Therefore we decided to use PCT as an additional diagnostic criterion for sepsis and septic shock. The cut-off value for PCT was > 0.5 ng/mL as it was proved that systemic infection is unlikely with concentration < 0.5 ng/mL^15^. We carefully selected the study subjects finally enrolled in the study. The first group of exclusion criteria were factors having impact on accuracy of ID/IDA diagnostics: bleeding, use of oral or parenteral iron in the last 3 months, RBC transfusion in the last 3 months, history or suspicion of thalassemia, macrocytosis, and pregnancy. Suspicion of thalassemia was based on the Mentzer index value < 13^16^. The Mentzer index is calculated as mean cell volume (MCV) expressed in fL divided by RBC expressed in millions per µL. Because MCV influences the Mentzer index result, macrocytosis was also listed as an exclusion criterion. Moreover macrocytosis falsely elevates Ret-He. Macrocytosis was defined as MCV above the local laboratory upper limit of reference range (i.e. > 96 fL). Only after exclusion of the aforementioned factors diagnosis of ID/IDA could be established accurately. The second group of exclusion criteria was absence of ID or IDA. The third group of exclusion criteria were contraindications to intravenous iron reported by the manufacturer: history of severe adverse event associated with parenteral iron, allergy to a constituent of intravenous iron complex, acute liver dysfunction, and acute hepatitis. The study flow chart in presented in Fig. 1.

### Clinical setting

In the local ICU every effort is taken to minimize the volume of blood lost for laboratory diagnostics. Laboratory tests are ordered only when necessary for diagnosis or management of patients. The lowest volume test tubes compatible with laboratory analyzers are used: 2 mL for complete blood count (CBC), 2.5 mL for biochemistry, 2.7 mL for coagulation profile. For point-of-care blood gas analysis 1 mL heparinized syringes filled to half volume are used. All blood samples are withdrew through an arterial cannula using a closed system, therefore there was no discarded blood during sampling. Although the volumes of blood used for laboratory diagnostics are minimal in the local ICU, we recorded volumes of withdrew blood in order to account for iatrogenic blood loss.

### Diagnosis of iron deficiency and iron deficiency anemia

Anemia was defined as Hb concentration < 120 g/L in women and < 130 g/L in men^17^. Diagnosis of ID/IDA was based on Ret-He and Hb concentration. Reticulocyte Hb equivalent allows for early diagnosis of ID/IDA, well before RBC indices become abnormal, and reflects current bone marrow iron availability^18^. Iron deficiency was diagnosed when Hb concentration was normal and Ret-He was below the lower limit of reference range (i.e. < 30.2 pg). Iron deficiency anemia was diagnosed when Hb concentration met WHO diagnostic criteria for anemia and Ret-He was below the lower limit of reference range. We decided to use the lower limit of Ret-He reference range provided by the manufacturer as various cut-off values for Ret-He were extrapolated from studies that utilized iron tests, e.g. transferrin saturation^19,20^. All hematological parameters were determined simultaneously using a single 2 mL EDTA test tube (BD Vacutainer, Becton Dickinson, United Kingdom) on a standard laboratory hematology analyzer (XN-1000, Sysmex, Japan). Ret-He determination was repeated after 2 and then after 4 doses of intravenous iron, on day 4 and 9.

### Intervention

Study subjects who were diagnosed with ID or IDA according to the abovementioned diagnostic criteria received iron dextran (CosmoFer, Pharmacosmos A/S, Denmark) in the recommended higher dose of 0.2 g intravenously (IV), three times a week, on days 1, 3, 5, 8, 10, 13. Generally, the safety of modern intravenous iron preparations (i.e. low molecular weight iron dextran, ferric carboxymaltose, ferric derisomaltose, iron sucrose, ferumoxytol) is comparable. The study subjects in our study were inpatients hospitalized in the ICU, therefore the duration of infusion was not an issue. As intravenous iron preparations are relatively costly, our choice was mainly dictated by the economic factors. As far as the dose of iron dextran is concerned, we took several factors into account. Firstly, as increase in Hb concentration following intravenous iron supplementation starts relatively late (maximal effect after 2 weeks), we aimed at relatively timely replenishment of iron stores, therefore we used a higher divided dose of iron dextran recommended by the manufacturer. Secondly, our intension was to use the lowest required dose of intravenous iron in order to not exceed binding capacity of transferrin and through this prevent deleterious effects of free iron and iron overload. Iron dextran was given as an intravenous drip, starting with a test dose (25 mg) given over 15 min. In case of adverse reaction the infusion had to be stopped immediately and appropriate measures be undertaken. Monitoring of iron supplementation was with Ret-He determined on day 4 and 9. Intravenous iron was continued until normalization of Ret-He. Irrespective of iron supplementation, study subjects with acute kidney injury^21^ or chronic kidney disease (estimated glomerular filtration rate < 60 mL/min before ICU admission) received epoetin alpha (Binocrit, Sandoz, Poland) in the recommended dose of 50 units/kg IV, three times a week, on the same days as iron dextran. We recorded daily volumes of blood lost for laboratory diagnostics to account for the possible effect of iatrogenic blood loos on the laboratory results.

### Statistical analysis

Statistical analysis was performed using MedCalc v.18 statistical software (MedCalc Software, Ostend, Belgium). Quantitative variables were expressed as medians and interquartile ranges (IQR, i.e. 25pc–75pc). The Shapiro–Wilk test was used to verify the type of distribution of quantitative variables. Qualitative variables were expressed as frequencies and percentages. The Wilcoxon signed-rank test for paired samples or the paired-samples t-test was used to determine between-group differences for quantitative variables before and after consecutive intravenous iron doses, depending on type of distribution. Between-group differences for quantitative variables were calculated with U-Mann–Whitney test or independent samples t-test, depending on type of distribution. All tests were two-sided. A *p* value < 0.05 was considered statistically significant.

This publication is part of the project tilted “Iron Metabolism Disorders in Patients With Sepsis or Septic Shock. Diagnosis and Monitoring of Treatment Based on Standard and New Laboratory Parameters”. The study was registered (1/02/2022) at ClinicalTrials.gov (identifier: NCT05217836), https://clinicaltrials.gov/ct2/show/NCT05217836.

The study was conducted in accordance with the Declaration of Helsinki.

### Ethics

This prospective interventional clinical study was approved by the Ethics Committee of the Medical University of Silesia in Katowice, Poland (PCN/CBN/0022/KB1/06/II/20/21, date of decision: June 29th 2021). Informed consent was obtained from all subjects involved in the study. The Consolidated Standards of Reporting Trials (CONSORT) Statement was applied for appropriate data reporting.

## Results

The qualified study population included 35 subjects. The median age in the study group was 69 (IQR 60–73) years. There were 15 (43%) women and 20 (57%) men in the study group, and their ages were 70 (IQR 65–77) and 69 (IQR 58–72) years, respectively. The median time of the ICU stay was 11 (IQR 7–21) days. Clinical characteristics of the study subjects are presented in Table 1. The study subjects were characterized by high severity of illness. The predicted in-hospital mortality rate was 20% (SOFA), 30–40% (APACHE II), and 50% (SAPS II). Above one third of the study group had acute kidney injury (AKI), and one fifth was receiving continuous renal replacement therapy. The most frequent anatomical site of infection were lungs, abdominal cavity, and urinary tract. The laboratory parameters of the study subjects are presented in Table 2. Study subject were characterized by high values of inflammatory markers (PCT, CRP). It is worth to stress that almost all erythrocyte indices were within reference range, with the exception of slightly elevated RDW-SD. Percentage and number of Rets were within normal values. As far as Ret subpopulations are concerned, IRF was highly elevated, LFR slightly decreased, MFR and HFR were normal.

**Table 1:** Clinical characteristics of the study subjects.

Characteristic	Value
APACHE II^a^, Me^b^, IQR^c^ (points)	23 (16–28)
SAPS II^d^, Me, IQR (points)	53 (37–68)
SOFA^e^, Me, IQR (points)	9 (7–12)
Acute lung injury (n, %)	0 (0)
Acute kidney injury (n, %)	13 (37)
Renal replacement therapy (n, %)	7 (20)
Chronic kidney disease (n, %)	4 (11)
Anatomical site of infection	
Pulmonary (n, %)	13 (36)
Abdominal (n, %)	8 (23)
Urinary (n, %)	8 (23)
Biliary (n, %)	2 (6)
Meningitis (n, %)	2 (6)
Other (n, %)	1 (3)
Unknown (n, %)	1 (3)
Outcome	
Survivors (n, %)	27 (77)
Deceased (n, %)	8 (23)

^a^Acute Physiology and Chronic Health Evaluation II.

^b^Median value.

^c^Interquartile range.

^d^Simplified Acute Physiology Score II.

^e^Sepsis Outcome Failure Assessment.

**Table 2:** Laboratory parameters of the study subjects.

Parameter	Value, Me^a^ (IQR)^b^	Reference range
Procalcitonin (ng/mL)	3.20 (1.23–20.78)	< 0.50
C-reactive protein (mg/L)	221 (131–274)	< 0.5
Aspartate aminotransferase (u/L)	56.4 (27.1–138.0)	< 35.0
Alanine aminotransferase (u/L)	27.4 (17.4–116.8)	< 35.0
Total bilirubin (mg/dL)	0.43 (0.28–0.73)	0.30–1.20
Creatinine (mg/dL)	1.72 (0.74–2.55)	0.50–0.90
Blood urea nitrogen (mg/dL)	41.5 (24.5–53.7)	7.8–20.0
RBC^c^ (10^6^/µL)	3.22 (2.95–3.98)	4.50–5.90
Hb^d^ (g/L)	96 (86–115)	140–175
Hematocrit (%)	30.9 (26.7–34.1)	40.0–52.0
Mean cell volume (fL)	90.4 (85.4–96.3)	80.0–96.0
Mean cell Hb (pg)	29.9 (27.9–31.1)	28.0–33.0
Mean cell Hb concentration (g/dL)	32.0 (31.3–33.5)	33.0–36.0
RBC distribution width-SD (fL)	50.9 (46.8–55.7)	35.3–50.3
RBC distribution width-CV (%)	15.0 (14.0–17.1)	10.0–15.0
Rets^e^ (%)	1.5 (1.1–2.1)	0.5–1.5
Rets (10^6^/µL)	0.05 (0.04–0.07)	0.04–0.12
Immature rets fraction (%)	18.3 (12.3–25.6)	1.0–8.9
Low fluorescence ratio rets (%)	81.7 (74.4–87.7)	84.4–96.5
Medium fluorescence ratio rets (%)	12.7 (10.3–15.8)	2.6–13.8
High fluorescence ratio rets (%)	3.5 (2.2–10.2)	0.0–6.3
Ret-He^f^ (pg)	27.2 (24.6–28.8)	30.2–36.2

^a^Median value.

^b^Interquartile range.

^c^Red blood cell.

^d^Hemoglobin.

^e^Reticulocytes.

^f^Reticulocyte hemoglobin equivalent.

**Figure 2:**
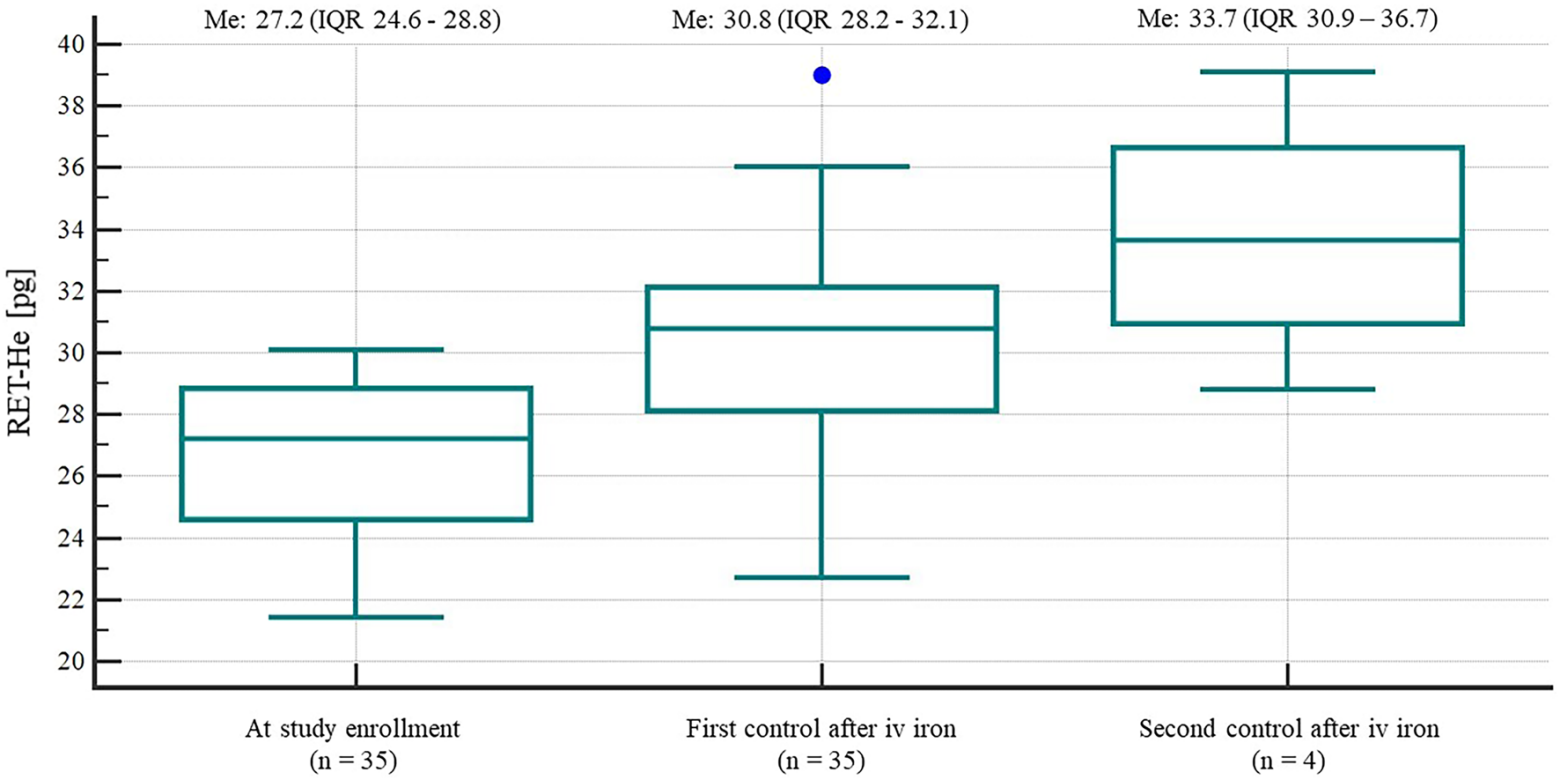
Reticulocyte hemoglobin equivalent at enrollment, on day 4 (first control) and 9 (second control) from the study enrollment.

Changes in Ret-He during intravenous iron supplementation are presented in Fig. 2. Median increase in Ret-He between successive determinations was 3.0 (IQR 1.9–6.1) pg. The change between first and second Ret-He determination (after 2 doses of iron dextran) was significant (*p* < 0.01), whereas between second and third Ret-He determination (after 4 doses of iron dextran) was not (*p* = 0.09).

The median time to Ret-He normalization was 4 (IQR 3–5) days. There were 35 (100%) study subjects who received iron dextran and 15 (43%) study subjects who received epoetin alpha additionally. The median dose of intravenous iron and epoetin alpha given to study subjects was 0.4 (IQR 0.4–0.6) g and 8000 (IQR 8000–16,000) units, respectively. The median time to the administration of the first iron dose (time from the ICU admission to the study enrollment) was 2 (IQR 2–3) days: 8 (23%) patients were enrolled day 1, 16 (45%) patients were enrolled on day 2, 4 (11%) patients were enrolled on day 3, 2 (6%) patients were enrolled on day 4, 1 (3%) subject was recruited on day 6, 2 (6%) subjects were enrolled on day 7, one patient (3%) was recruited on day 8 and one patient (3%) reached the inclusion criteria on day 19 from the ICU admission.

Analysis of subjects who received iron dextran (n = 20) and those who received both iron dextran and epoetin alpha (n = 15), showed no significant between-group differences in Ret and erythrocyte parameters determined throughout iron supplementation (Table 3). There were also no meaningful differences between subpopulations of patients diagnosed with or without acute kidney injury, with or without chronic kidney disease in terms of erythrocyte and reticulocytic parameters. Also, renal replacement therapy did not influence the considered reticulocytic parameters (*p* > 0.05 for between-group analyzes).

**Table 3:** Laboratory parameters of the study subjects divided into subgroups of patients receiving iron dextran or both iron dextran and epoetin alpha.

Parameter	Iron dextran (n = 20), value, Me^a^ (IQR)^b^	Iron dextran and epoetin alpha (n = 15), value, Me^a^ (IQR)^b^	*p*
Procalcitonin (ng/mL)	3.07 (1.10–15.9)	5.17 (1.86–25.21)	0.39
C-reactive protein (mg/L)	224 (134–262)	217 (114–305)	0.63
Aspartate aminotransferase (u/L)	53 (29–136)	81 (26–194)	0.62
Alanine aminotransferase (u/L)	30 (18–108)	27 (17–210)	0.89
Total bilirubin (mg/dL)	0.46 (0.28–0.76)	0.40 (0.28–0.55)	0.56
Creatinine (mg/dL)	1.09 (0.69–2.17)	2.12 (1.53–3.00)	0.06
Blood urea nitrogen (mg/dL)	41.6 (24.6–48.9)	41.5 (25.1–56.2)	0.69
RBC^c^ (10^6^/µL)	3.35 (2.96–3.89)	3.19 (2.92–4.03)	0.92
Hb^d^ (g/L)	97 (87–114)	96 (84–114)	0.62
Hematocrit (%)	31 (27–34)	30 (26–34)	0.64
Mean cell volume (fL)	90.9 (87.5–96.9)	90.0 (83.9–96.1)	0.55
Mean cell Hb (pg)	29.9 (28.5–31.1)	29.0 (26.7–31.4)	0.93
Mean cell Hb concentration (g/dL)	32.2 (31.3–33.4)	32.0 (31.6–33.7)	0.88
RBC distribution width-SD (fL)	49.4 (45.5–56.3)	53.2 (49.6–55.7)	0.49
RBC distribution width-CV (%)	14.6 (14.0–17.2)	16.4 (13.8–17.0)	0.82
Rets^e^ (%)	1.41 (0.91–2.15)	1.51 (1.22–2.08)	0.83
Rets (10^6^/µL)	0.048 (0.036–0.096)	0.050 (0.039–0.063)	0.75
Immature rets fraction (%)	18.2 (11.9–26.0)	18.5 (12.4–24.2)	0.93
Low fluorescence ratio rets (%)	81.8 (74.0–88.2)	81.5 (75.9–87.6)	0.91
Medium fluorescence ratio rets (%)	14.1 (9.5–15.7)	12.2 (10.4–15.9)	0.75
High fluorescence ratio rets (%)	3.55 (2.05–10.20)	3.50 (2.33–9.30)	0.91
Ret-He^f^ (pg)	27.0 (24.7–29.0)	27.3 (24.4–28.6)	0.96

^a^Median value.

^b^Interquartile range.

^c^Red blood cell.

^d^Hemoglobin.

^e^Reticulocytes.

^f^Reticulocyte hemoglobin equivalent.

Reticulocyte subpopulations and their changes following iron supplementation are presented in Table 4.

All Ret subpopulations changed significantly after two doses of iron dextran (*p* for all < 0.01): increased IRF rose sharply, slightly decreased LFR decreased further, normal MFR and HFR increased above the upper limit of reference range. However neither percentage of Rets, nor number of Rets, changed significantly following iron dextran.

Regarding erythrocyte indices, significant increase following iron supplementation was noted for MCV, RBC distribution width-SD (RDW-SD), and RBC distribution width-CV (RDW-CV), and RBC. Decrease in mean cell hemoglobin concentration (MCHC) was noted. Change in Hb concentration was not significant as well as remaining CBC parameters (MCH; HCT) (Table 4).

**Table 4:** Differences in erythrocyte and reticulocytic indices before the administration of intravenous iron dextran and after two doses of iron dextran.

Parameter	Before IV iron administration (n = 35) Value, Me^a^ (IQR)^b^	After IV iron administration (n = 35) Value, Me^a^ (IQR)^b^	*p*
RBC^c^ (10^6^/µL)	**3.22 (2.95–3.98)**	**3.24 (2.76–3.84)**	**0.03**
Hb^d^ (g/L)	96 (86–115)	96 (86–111)	0.05
Hematocrit (%)	30.9 (26.7–34.1)	30.3 (25.5–34.6)	0.18
Mean cell volume (fL)	**90.4 (85.4–96.3)**	**93.4 (87.1–98.2)**	** < 0.01**
Mean cell Hb (pg)	29.9 (27.9–31.1)	29.9 (28.1–31.1)	0.92
Mean cell Hb concentration (g/dL)	**32.0 (31.3–33.5)**	**31.6 (30.5–32.5)**	** < 0.01**
RBC distribution width-SD (fL)	**50.9 (46.8–55.7)**	**53.2 (49.9–57.5)**	** < 0.01**
RBC distribution width-CV (%)	**15.0 (14.0–17.1)**	**15.3 (14.5–17.8)**	** < 0.01**
Rets^e^ (%)	1.51 (1.15–2.09)	1.42 (1.06–2.37)	0.39
Rets (10^6^/µL)	0.049 (0.039–0.066)	0.047 (0.030–0.065)	0.88
Immature rets fraction (%)	**18.3 (12.3–25.6)**	**29.3 (20.8–38.7)**	** < 0.001**
Low fluorescence ratio rets (%)	**81.7 (74.4–87.7)**	**70.7 (61.3–79.2)**	** < 0.001**
Medium fluorescence ratio rets (%)	**12.7 (10.3–15.8)**	**16.0 (12.7–20.4)**	** < 0.01**
High fluorescence ratio rets (%)	**3.5 (2.2–10.2)**	**13.0 (4.9–18.7)**	** < 0.001**
Ret-He^f^ (pg)	**27.2 (24.6–28.8)**	**30.8 (28.2–32.1)**	** < 0.0001**

Significant values are in [bold].

^a^Median value.

^b^Interquartile range.

^c^Red blood cell.

^d^Hemoglobin.

^e^Reticulocytes.

^f^Reticulocyte hemoglobin equivalent.

**Figure 3:**
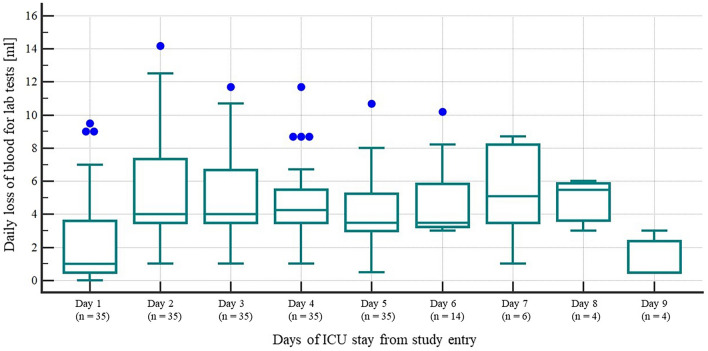
Daily blood volume lost for laboratory diagnostics in the study group.

Daily volume of blood lost for laboratory diagnostics in the study subjects was negligible (Fig. 3).

In the study group follow-up investigation we determined the in-hospital mortality and general further fate of our study subjects. Among patients included in our study, eight subjects (n = 8/23%) died during the ICU stay, two subjects (n = 2/6%) died during further hospitalization outside of the ICU, and twenty-five subjects (n = 25/71%) were discharged from the hospital. The median time of the hospital stay in our medical center after the ICU discharge was 4 (IQR 0–15) days. Survivors (n = 27/77%) were discharged to on-site and external locations. The on-site locations were the following departments: internal medicine (n = 3), gastrointestinal surgery (n = 3), neurology (n = 2), gastroenterology and hepatology (n = 2), neurosurgery (n = 2), oncological surgery (n = 1), gynecology and obstetrics (n = 1), and neurological rehabilitation (n = 1). The external locations were as follows: internal medicine department (n = 5), palliative care centre (n = 2), cardiology department (n = 2), ICU (n = 1), neurology department (n = 1), and nephrology department (n = 1).

## Discussion

In the presented study we intended to examine the impact of intravenous iron supplementation on Ret-He and Ret subpopulations in iron-deficient patients with sepsis or septic shock hospitalized in the ICU. In our study we used an established international definition of sepsis and septic shock^13^. Determination of Ret-He, compared to standard iron tests, offers more precision and sensitivity in determination of ID in the ICU, as it has been shown in one of the recent publications^22^. The analyzer that was used in this research project was a standard analyzer used on a daily basis in our medical center’s central laboratory, the determination of Ret parameters was not outsourced. Our intension was to use a diagnostic method that is relatively widely available. It is a well-known fact that clinicians may not be aware of possibility to run a full Ret analysis on current hematology analyzers. Standard CBC result does not include Ret parameters—a separate order is required.

Diagnostic gold standard for ID is absence of stainable bone marrow^23^. The method looks for non-heme iron in erythroblasts and extracellular space using the Prussian blue staining and potassium ferrocyanide in a bone marrow sample. The fraction of erythroblasts containing iron (sideroblasts) reflects amount of iron incorporated in these precursor cells. The fraction of sideroblasts reflects functional availability of iron for erythropoiesis^24^. With this method it is feasible to distinguish between functional ID (FID, iron in extracellular space and low sideroblasts) and absolute ID (AID, no iron in extracellular space and low sideroblasts)^25^. However this method is an invasive procedure, with a relatively long turn-around time, mostly performed in non-critically ill patients hospitalized in medical centers specializing in hematology, therefore routine aspiration of the bone marrow sample is rarely performed, reserved for complex cases^26^. There was search for other means to diagnose iron depletion that avoid risks coming from bone marrow aspiration^26^. Biomarkers have been tested to determine which of them best correlate with stainable bone marrow iron^27^. Serum ferritin proved to be the pillar indicator of iron stores and the most sensitive and specific biomarker for assessing ID^28^. We did not use the standard iron tests in the study, as it has been shown that they are not reliable for diagnosis of ID in the critically ill patients presenting with systemic inflammation. The authors analyzed the matter in the previous publication^29^. Other valuable biomarkers turned out to be hepcidin and soluble transferrin receptor (sTFR)^30^. Hepcidin concentration is usually low or normal in absolute ID and it helps distinguish AID from FID. The sTFR is a valuable indicator of ID because unlike ferritin it is unaffected by inflammation^31^. In our study we used a relatively new reticulocyte parameter, Ret-He, which is available on new hematology analyzers and have some advantages over the previously mentioned parameters.

Iron deficiency is one of the most frequent deficiencies worldwide. It is mainly due to relatively low intake with unbalanced diet and no supplementation^32^. Setting the ground for IDA prevalence in general population, critically ill patients hospitalized in the ICU are endangered by aggravation of the disorder or its development de novo. Critically ill patients may require parenteral nutrition, however standardized intravenous formulas may not contain trace elements due to problems with stability in solution^31^. In order to supplement trace elements with parenteral formulas additional solutions are needed, however these may be omitted because of lack of knowledge^32^. Sepsis patients are at high risk of malnutrition and frequently require total or supplemental parenteral nutrition, for instance when the source of sepsis is located within the abdominal cavity or enteral food intake is impossible due to a gastrointestinal tract disorder^33^. Nonetheless, trace elements supplementation, such as iron, is still debatable in states like sepsis or cardiac surgery^34^. It was shown that iron concentration decreases during SIR and this induced ID may be partially obscured by increase of ferritin concentration^35^. The American Society of Parenteral and Enteral Nutrition guidelines reported conflicting results regarding the influence of iron supplementation on mortality, secondary infection, time on vasopressors or mechanical ventilation, hospital length of stay in sepsis^36^. The guidelines suggest to provide micronutrients supplementation, including iron, to critically ill patients, particularly burn and trauma patients, patients requiring mechanical ventilation, exception being sepsis patients^36^. Lack of iron supplementation in sepsis patients may lead to ID or even IDA, what may expose these patients to RBC transfusion with transfusion-related adverse events and complications. Some of these complications may be potentially fatal, for instance transfusion-related acute lung injury (TRALI)^37^. Moreover, sepsis patients may require renal replacement therapy (RRT), during which microelements, such as iron, are removed with the effluent^38^.

The presented study shown that iron supplementation with divided doses of iron dextran in iron-deficient sepsis patients relatively quickly normalizes Ret-He. After only two doses of iron dextran, within 4 days, Ret-He normalized in almost all study subjects. In our study change in Hb concentration was not statistically significant. This is not surprising. Effect of intravenous iron on Hb concentration is delayed due to RBC lifetime (120 days), with notable increases in Hb concentration seen after minimum 2 weeks. We noticed significant changes in all Ret subpopulations. Immature Rets fraction which was highly elevated at baseline increased even further, whereas MFR and HFR which were normal at baseline increased above the upper limit of reference ranges, LFR slightly decreased at baseline decreased further. As the degree of fluorescence is proportional to the amount of RNA in the Rets, changes in Ret subpopulations following iron dextran suggest increased erythropoietic activity. Our study suggests that IV iron supplementation may be effective in sepsis patients with ID or IDA. Intravenous iron supplementation could potentially decrease the requirement for allogeneic RBC transfusion in sepsis patients hospitalized in the ICU. It is important to avoid RBC transfusion in the population of sepsis patients, as it may lead to disturbed microcirculation caused by RBC storage lesion. Such effects were widely examined by Ferrara et al. who observed reperfusion microvascular injury after massive transfusions in the animal model of hemorrhagic shock^39^. The researchers obtained the micro-videoscopic images of the intestinal mucosa and serosa, and sidestream-dark-field imaging of the sublingual mucosa. Their observations indicate that despite normalization of aerobic metabolism parameters, microvascular reperfusion injury was present in all three studied vascular beds following transfusion. The recovery of microvascular perfusion was incomplete, and regional deficits persisted^39^.

Another issue is the safety of intravenous iron supplementation, particularly in the population of patients already burdened with multi-organ dysfunction syndrome and acute organ injury resulting from sepsis or septic shock. In our study the predicted in-hospital mortality rate was 20–50%, the observed mortality rate was close to the lower limit of this range—23%. There were no side effects reported by the manufacturer in the study group. We did not analyze any other adverse effects of intravenous iron complex used in the study. Clark et al. investigated the effect of intravenous iron in AKI patients, pointing out that anemia may be even more widespread among patients with AKI, where endogenous erythropoietin (Epo) production is usually impaired^40^. Intravenous iron is well-known to optimize anemia and minimize Epo use in chronic kidney disease (CKD) population^41–44^. There are however controversies regarding the use of intravenous iron in AKI patients as there is paucity of data regarding its risk–benefit profile, moreover excess free iron has been associated with increased oxidative stress and adverse cardiovascular events and outcomes^45,46^. There are no established guidelines regarding intravenous supplementation of iron in AKI patients. Iron stores may not be controlled in a particular patient until the development of anemia resistant to increasing doses of Epo, besides serum iron markers may be difficult to interpret due to the fact that ferritin is an acute-phase reactant which may disrupt the interpretation of ID in CKD patients^47,48^. Recent research by Ishida et al. attempted to confirm the safety of intravenous iron in patients with impaired renal function admitted for bacterial infection^49^. The authors showed no association between intravenous iron and higher 30-day mortality, longer length of stay or increased risk of readmission within 30 days^49^. Clark et al. retrospectively analyzed 134 patients diagnosed with AKI, 67 patients who received intravenous iron and 67 controls. The authors concluded that there were no adverse consequences of intravenous iron used to treat resistant anemia in patients with AKI—no negative effect of intravenous iron on recovery of AKI or mortality was seen, not even in sepsis inpatients receiving antibiotics^40^. We feel that there is room for further research regarding risk–benefit of intravenous iron supplementation in patients with sepsis or septic shock. The recent meta-analysis from 2021 reported increased risk of infection associated with intravenous iron compared to oral iron or no iron based on evidence of moderate certainty. The relative risk (RR) was 1.16 (95% CI 1.03–1.29; moderate heterogeneity I^2^ = 36%, *p* = 0.003). After exclusion of studies with high risk of bias, risk of infection associated with intravenous iron was not statistically significant [RR 1.13 (95% CI 0.97–1.32); I^2^ = 36%; *p* = 0.08]. The authors showed Hb increase associated with intravenous iron (mean difference, 5.7 g/L; 95% CI 5.0–6.4 g/L; I^2^ = 94%, *p* < 0.001) and decrease in risk of RBC transfusion (RR 0.93, 95%CI 0.76–0.89, I^2^ = 15%, *p* < 0.001), compared no iron or oral iron. The authors concluded that further research with standardized definition of infection are required to fully explore the risk–benefit profile of intravenous iron^50^. Finally, one of the latest study on iron status in sepsis was published at the beginning of 2023 authored by Hamilton et al. The authors preformed an observational study with Mendelian randomization to test the hypothesis that increasing levels of iron biomarkers increase the risk of sepsis. They showed through stratified analyzes, that sepsis risk may be larger in subjects with iron deficiency and/or anemia. The authors elaborated upon ferritin being an iron storage protein generally considered a biomarker of iron body stores in a non-inflammatory state. Although this particular project focused more on the classic iron markers than reticulocytic parameters, it underlines the growing interest in the vast research area that concerns iron supplementation^51^.

Our study is by no means free of limitations. Firstly, although we screened all consecutive patients with sepsis or septic shock during 12 month period, the study group was relatively small. This was due to the fact that only sepsis patients with true ID or IDA (based on Ret-He) were enrolled in the study, which is a strong feature of the study. Only patients with ID may benefit from supplementation, therefore studies in which intravenous iron was administered indiscriminately might not show the true effects of the medication^52^. Although the study group was small, it was numerous enough for the statistical analysis to reach significance. Moreover, we performed additional a posteriori power calculations regarding our project’s sample size for the study group, in order to increase the reliability of our results. We discovered that we would require a minimum of 32 pairs to verify intravenous iron effect with an alpha of < 0.0001 and a beta of 0.20. Therefore, we acknowledge that our sample size is sufficient to draw conclusions, and our study was in no way underpowered. Secondly, we acknowledge that the diagnostic gold standard for ID/IDA diagnostics is staining of a bone marrow sample, however it not practical^23–25^, and in our view could be seen unethical in the critically ill. Thirdly, we acknowledge that we did not examine whether changes in Ret-He and other Ret parameters translate into changes in Hb concentration, however it was not the aim of our study. In order to show differences in Hb concentration the study subjects had to be followed for a longer time period (maximal effect of intravenous iron after 2 weeks), and the aim of our study was to show the acute effects of intravenous iron, administered only until normalization of Ret-He, so iron overload was prevented.

## Conclusions

Iron supplementation with divided doses of iron dextran in iron-deficient sepsis patients relatively quickly normalizes Ret-He. Changes in Ret subpopulations suggest increased erythropoietic activity. Further research is needed to explore the role of intravenous iron in this clinical scenario.

## Data Availability

The datasets used and/or analyzed during the current study are available from the corresponding author on reasonable request.
